# The Attributable Factors That Increase the Likelihood of Central Line Associated Blood Stream Infection Related In-Hospital 30-Day Mortality

**DOI:** 10.7759/cureus.35898

**Published:** 2023-03-08

**Authors:** Naif H Alotaibi, Abdulrahman M Barri, Ali M Somily

**Affiliations:** 1 Internal Medicine Department, College of Medicine, King Saud University, Riyadh, SAU; 2 Microbiology Department, King Saud University, Riyadh, SAU

**Keywords:** mortality, central line associated infection, clabsi, 30 days mortality, in-hospital mortality

## Abstract

Objective: The objective is to investigate the attributable factors associated with an increase in hospital 30-day mortality of central line bloodstream infection (CLABSI).

Methods: A retrospective cohort study was conducted at King Saud University Medical City (KSUMC). The sample included adult patients who developed CLABSI between March 2016 and February 2018 after having a central line inserted at KSUMC in Riyadh, Saudi Arabia.

Results: A total of 283 patients were involved in the study. The 30-day mortality rate was 18.8%. Patients were more likely to die if they were in the intensive care unit (ICU) or required ICU admission after infection (p<0.001). This was also observed in patients who required inotropes or intubation before or after culture (p<0.001). There was a statistically significant difference of 6.60±5.62 in the mean score on the Acute Physiologic Assessment and Chronic Health Evaluation (APACHE) II between before and after culture (p<0.001). The likelihood of death was significantly higher among patients with higher APACHE II scores before and after culture (p<0.001). The presence of CLABSI-related sequelae was not associated with increased mortality (p<0.595).

Conclusions: The clinical characteristics of CLABSI patients are variable and can increase the risk of mortality or complicate the treatment course. Physicians should be aware of the significance of these factors as potential causes of increased mortality.

## Introduction

A central venous catheter (CVC) is an intravascular device used for dialysis or to deliver potentially irritating medications, such as vasopressors and chemotherapeutic agents, to large caliber vessels, as well as nutritional support. These lines are usually used to manage hemodynamically unstable patients, but they also carry the risk of promoting hospital-acquired infections. This poses a substantial threat to patient safety and increases mortality risk [1]. Central line-associated bloodstream infection (CLABSI) is defined by the Centers for Disease Control (CDC) and Prevention as “a laboratory-confirmed bloodstream infection (LCBI) where an eligible BSI organism is identified, and an eligible central line is present on the LCBI day of an event or the day before” [2].

CLABSI is a common, preventable, hospital-acquired infection with approximately 250,000 new cases in the United States each year [3]. It is especially common in critically ill patients in intensive care units (ICUs) where it carries a risk of mortality [4]. However, prolonged periods of central line use may lead to more severe disease, which can also influence mortality [5]. In previous studies, CLABSI has been associated with increased hospital costs and length of stay [6,7]. The risk of developing CLABSI can be reduced through infection control measures designed to improve hand hygiene practices and catheter care [8]. These measures are lacking in developing countries due to limited resources and a lack of awareness, which contribute to higher rates of infection [9].

## Materials and methods

Approval was obtained from the King Saud University institutional review board before starting the study, reference number 18/0373/IRB. The study was also conducted according to the principles of the Helsinki Declaration. This retrospective cohort study examined patient records between March 2016 and February 2018 at King Saud University Medical City (KSUMC), Riyadh, Saudi Arabia. The 30-day mortality rate and relevant factors associated with increased mortality were examined. Patients were included if they were >18 years old, had a central line of any type inserted at KSUMC, and developed CLABSI, according to the definition of the CDC [2]. Patients were excluded if their central line was inserted at another institution, even if they presented with CLABSI at KSUMC. A list of all positive cultures from central lines was obtained from the archives of the microbiology lab. The list was filtered to include only patients with an eligible BSI organism and an eligible central line. 

Definitions

Laboratory-confirmed bloodstream infection (LCBSI) was defined by two criteria. Criterion 1 was that the identified organism, which is always recognized as a pathogen, is isolated from one or more blood cultures that are unrelated to an infection at other sites. Criterion 2 was that the isolation of common skin commensal organisms, such as coagulase-negative Staphylococcus, from two or more blood cultures taken on different occasions, along with at least one of the following: chills, fever (temperature greater than 38° C) or hypotension, and the identified organism is not related to an infection at other sites [2]. An eligible central line has been in place for two or more consecutive days following the first access of the line until 1 day after line removal or patient discharge [2]. CLABSI prevention bundle is a checklist based on the 2011 CDC guideline for the prevention of intravascular catheter-associated bloodstream infections [10].

Data collection

After a brief training on the CDC CLABSI diagnostic criteria, electronic medical records were checked to collect information on demographics, admission characteristics and length of stay, central line characteristics, where was inserted and whether CLABSI prevention bundle use was documented, causative organisms, treatment, clinical outcomes of episodes and if developed a Sequele, and whether inotropes or intubation was required. Central lines were categorized as permanent (tunneled dialysis catheter) or temporary non tunneled non implanted catheter) catheters. patients were considered to have a temporary CVC if there two lines were present, and one of them was a temporary line.

Statistical analysis 

Statistical analyses were performed using SPSS version 22.0 (IBM Corp., Armonk, NY, USA). Demographics and 30-day mortality related to the central line were considered since mortalities beyond 30 days were regarded as less likely to be related to the central line infection. Categorical data were expressed as frequencies and percentages, and continuous data were expressed as mean and standard deviation (SD). Values associated with increased mortality risk were identified using the chi-squared test and an independent-sample t-test as appropriate. The P-value was considered significant when ≤ 0.05.

## Results

A total of 283 patients were enrolled, and more than 50% were male (157 patients [55.5%]). The mean age of the patients was 52.8±20.5 years with a range of 14-90 years. 52 patients (18.4%) were admitted due to CLABSI. Approximately half had ICU admissions either before CLABSI or because of it (138 patients (48.8%)), and half of them were in the surgical ICU (SICU; 69 patients [50.4%]; Table 1). The number of patients who required inotropic support after developing CLABSI increased compared to pre-infection, and some patients required higher inotropic support compared to previous requirements. The number of patients who required intubation increased only slightly after culture compared to pre-infection, and 31.9% required higher than previous FiO2 requirements (Table 1).

**Table 1:** Clinical characteristics compared by mortality SD: standard deviation, ICU: intensive care unit, CLABSI: central line-associated blood stream infection, HD: hemodynamic. Chi-square and independent t-test used

Variables	Value	30-day mortality N (%)		P-value
		Yes	No	
Age	Mean (SD)	63.25±18.14	50.16±20.31	0.000*
Gender	Male	31 (51.7)	126 (56.5)	0.559
	Female	29 (48.3)	97 (43.5)	
ICU admission	Yes	49 (35.5)	89 (64.5)	0.000*
	No	11 (7.6)	134 (92.4)	
Inotrope before CLABSI	Yes	23 (57.5)	17 (42.5)	0.000*
	No	37 (15.2)	206 (84.8)	
Inotrope after CLABSI	Yes	48 (49.0)	50 (51.0)	0.000*
	No	12 (6.5)	173 (93.5)	
Inotrope requirement	Increased	19 (63.3)	11 (36.7)	-
	Decreased	2 (40.0)	3 (60.0)	
	Same	2 (50.0)	2 (50.0)	
	Not required before culture	25 (42.4)	34 (57.6)	
Intubation before CLABSI	Yes	33 (35.1)	61 (64.9)	0.000*
	No	27 (14.3)	162 (85.7)	
Intubation after CLABSI	Yes	45 (39.1)	70 (60.9)	0.000*
	No	15 (8.9)	153 (91.1)	
Intubation requirement	Increased	15 (4.7)	21 (58.3)	-
	Decreased	2 (33.3)	4 (66.7)	
	Same	13 (28.9)	32 (71.1)	
	Not required before culture	13 (50.0)	13 (50.0)	

The mean age of patients who died was significantly (p<0.0001) higher than those who survived. The likelihood of death was higher in patients who were in the ICU or required ICU admission after infection (p<0.0001) (Table 1). There was no statistically significant (p<0.559) association between death and gender (Table 1). Patients who required inotropes or needed intubation before or after culture were more likely to die (p<0.0001; Table 1).

Most central lines were inserted by interventional radiology (178 patients (57.98%)). The use of CLABSI prevention bundles is standard practice, it was not documented for most patients for lines maintenance in 194 patients (68.6%; Table 2). Most patients had only a single central line (247 patients [87.3%]), 179 patients (63.3%) predominantly had permanent central lines, and 13 patients (6.57%) had their lines changed using a guidewire after CLABSI developed (Table 2).

**Table 2:** Central line characteristics Chi-square test used

Variables	Value	30-day mortality		P-value
		Yes	No	
Bundle maintenance	Yes	30	59	0.0001
	No	30	164	
Number of central line	Single	42	205	0.0001
	Multiple	18	18	
Type of central line	Permanent	16	163	0.0001
	Temporary	44	60	
Usage of guide wire to replace the line	Yes	1	12	0.194
	No	41	121	

Most patients were discharged without complications (75.5% [208 patients]), but 52 died within 30 days of infection (18.8%) and several suffered from CLABSI-related complications (5.8% [16 patients]). These complications included organ failure, hematologic derangement, and seeding of infection to another place. However, the presence of sequelae was not statistically (p<0.595) associated with increased mortality (Table 3).

**Table 3:** End point of patient

Variables	Value	N(%)
End point	Discharge	208(75.4%)
	Sequele	16(5.8%)
	Deaths	52(18.8)

The mean time of death after CLABSI was 8.1±7.5 days, and the time of removal of central lines after infection was 3.61±4.95 days (Table 4). Among patients who died, the mean number of days until developing CLABSI after a central line insertion was significantly (p<0.0001) lower than those who survived (Table 4).

**Table 4:** Central line characteristics compared to mortality CLABSI: central line-associated blood stream infection. Independent t-test used

Variables	Death N (%)			P-value
	Yes	No		
Mean #days to have CLABSI after line insertion	16.40±17.58		79.59±179.05	0.000*
Mean #days to have CLABSI after admission	36.50±31.16		71.89±277.24	0.066
Mean #days to death after developing CLABSI	8.07±7.49		-	-
Mean #days to have line removal after CLABSI	1.06±2.17		4.13±5.17	0.000*

## Discussion

Hospital-acquired infection is a growing burden worldwide [11]. Evidence suggests that the incidence of CLABSI-related complications is expected to increase in the future. However, effective infection-control programs may alleviate this [8]. The lack of awareness regarding CLABSI in resource-limited countries can greatly affect the number of cases that occur overall [9].

Most related studies on mortality have focused exclusively on ICU patients. Instead, our study involved all adult patients in a tertiary hospital, regardless of whether they were in the ICU or general wards. The rate of CLABSI-related 30-day mortality was 18.8% (52 patients) in our study, which is lower than the rates observed in a tertiary hospital in New York (28.4% [56 patients]) but comparable to a study undertaken in ICUs in Ecuador (18.5%) [4,12]. Mortality was found to be significantly higher among ICU patients and critically ill patients requiring hemodynamic support or respiratory mechanical support.

The need for prolonged central line days may suggest a more severe disease, which could also influence mortality [5]. However, this was not the case in our study, where the mean number of central line days of those who died was significantly lower than those who did not.

Multimodal approaches for CLABSI prevention have emerged around the world, consisting of multiple strategies [13]. The lack of adherence to these measures could represent a serious problem. The concept of CLABSI prevention bundles at the time of insertion, as well as for surveillance, was recently introduced with the aim of minimizing CLABSI incidence. In our study, CLABSI prevention bundles were seldom used at the time of central line insertion, and they were not used for maintenance in 194 patients (68.6%). Since the CLABSI prevention bundles used at the time of insertion were recorded with paper-based documentation during the study period, this could reflect a false-positive number. However, a strict obligation to use the documentation bundle at the time of insertion in electronic medical records started after the study period.

Study limitations

The heterogeneity of the participants is a potential limitation. The participants were recruited from different settings, including ICUs and general wards, and each had different characteristics and levels of mortality risk. Subgroup analysis is needed to focus on the specific risk of higher mortality in each subgroup. Additionally, our study did not estimate the incidence of developing CLABSI in all patients who received a central line.

## Conclusions

This study found that many of the patient’s clinical characteristics are associated with increased mortality of CLABSI in especially in critically ill patients. We believe that these findings add evidence to support measures aiming to reduce these infections and targeting the factors that are associated with increased mortality in different hospital settings.
